# Supplementary material for: The effect of long-range linkage disequilibrium on allele-frequency dynamics under stabilizing selection
Source: PLoS Genet. 2026 Mar 9;22(3):e1012035. doi: 10.1371/journal.pgen.1012035 (PMC12991805; doi:10.1371/journal.pgen.1012035)
Supplement: S1 Text — (PDF) [file pgen.1012035.s001.pdf]

S1 Text. Supplementary information for:

The effect of long-range linkage disequilibrium on allele-frequency dynamics under stabilizing selection

Sherif Negm, Carl Veller

S1 The average fitness of an allele, accounting for phenotypic variance

Suppose that an allele's trait distribution is normal with mean μ and variance V_P. To find the average fitness of this allele, we integrate the fitness function $e^{-y^2/2V_S}$ over the allele's trait distribution[1]:

$$
\begin{aligned}
\bar{w}(\bar{y}=\mu,\sigma^2=V_P) &= \int \overbrace{\frac{1}{\sqrt{2\pi V_P}}e^{-\frac{(y-\mu)^2}{2V_P}}}^{\text{trait distribution}} \cdot \overbrace{e^{-\frac{y^2}{2V_S}}}^{\text{fitness}}\,dy \\
&= \frac{1}{\sqrt{2\pi V_P}}\int \exp\left(-\frac{V_S(y-\mu)^2+V_Py^2}{2V_SV_P}\right)dy \\
&= \frac{1}{\sqrt{2\pi V_P}}\int \exp\left(-\frac{(V_S+V_P)y^2-2V_S\mu y+V_S\mu^2}{2V_SV_P}\right)dy \\
&= \frac{1}{\sqrt{2\pi V_P}}\int \exp\left(-\frac{y^2-2\frac{V_S\mu}{V_S+V_P}y+\frac{V_S\mu^2}{V_S+V_P}}{\frac{2V_SV_P}{V_S+V_P}}\right)dy \\
&= \frac{1}{\sqrt{2\pi V_P}}\int \exp\left(-\frac{\left(y-\frac{V_S\mu}{V_S+V_P}\right)^2+\frac{V_S\mu^2}{V_S+V_P}-\frac{V_S^2\mu^2}{(V_S+V_P)^2}}{\frac{2V_SV_P}{V_S+V_P}}\right)dy \\
&= \frac{1}{\sqrt{2\pi V_P}}\int \exp\left(-\frac{\left(y-\frac{V_S\mu}{V_S+V_P}\right)^2+\frac{V_S\mu^2}{V_S+V_P}\left(1-\frac{V_S}{V_S+V_P}\right)}{\frac{2V_SV_P}{V_S+V_P}}\right)dy \\
&= \frac{1}{\sqrt{2\pi V_P}}\int \exp\left(-\frac{\left(y-\frac{V_S\mu}{V_S+V_P}\right)^2+\frac{V_SV_P\mu^2}{(V_S+V_P)^2}}{\frac{2V_SV_P}{V_S+V_P}}\right)dy \\
&= \frac{1}{\sqrt{2\pi V_P}}\int \exp\left(-\frac{\left(y-\frac{V_S\mu}{V_S+V_P}\right)^2}{\frac{2V_SV_P}{V_S+V_P}}-\frac{\mu^2}{2(V_S+V_P)}\right)dy \\
&= \sqrt{\frac{V_S}{V_S+V_P}}e^{-\frac{\mu^2}{2(V_S+V_P)}}\cdot\int \frac{1}{\sqrt{2\pi\frac{V_SV_P}{V_S+V_P}}}\exp\left(-\frac{\left(y-\frac{V_S\mu}{V_S+V_P}\right)^2}{\frac{2V_SV_P}{V_S+V_P}}\right)dy \\
&= \sqrt{\frac{V_S}{V_S+V_P}}e^{-\frac{\mu^2}{2(V_S+V_P)}}, \qquad \text{(S.1)}
\end{aligned}
$$

[1]The result of this calculation is standard in the literature; we detail the calculation here only for expository purposes.

where the cancellation in the last line follows from the fact that the integrand in the second-to-last line is the probability density function of a normally distributed random variable [with mean $V_S\mu/(V_S+V_P)$ and variance $V_S V_P/(V_S+V_P)$]. Since we are interested only in relative fitness, and because the phenotypic variance is the same for the two alleles at the focal locus, we can ignore the constant factor at the front of Eq. (S.1) and write the relative fitness of the allele with trait mean μ and variance V_P as

$$\bar{w}(\bar{y}=\mu,\sigma^2=V_P)=e^{-\frac{\mu^2}{2(V_S+V_P)}}. \tag{S.2}$$

S2 Predicting the change in the genic and genetic variance from predictions of allele-frequency change

The expected change in the genic variance is

$$\mathbb{E}\left[\Delta V_g\right]=\mathbb{E}\left[\Delta\sum_l 2p_l(1-p_l)\alpha_l^2\right]=\sum_l 2\alpha_l^2\mathbb{E}\left[\Delta\{p_l(1-p_l)\}\right]. \tag{S.3}$$

Writing p_l' as the frequency of the focal allele at locus l next generation,

$$\begin{aligned}\mathbb{E}\left[p_l'(1-p_l')\mid p_l\right]&=\mathbb{E}\left[(p_l+\Delta p_l)(1-p_l-\Delta p_l)\mid p_l\right]\\&=p_l(1-p_l)+(1-2p_l)\mathbb{E}[\Delta p_l\mid p_l]-\mathbb{E}\left[(\Delta p_l)^2\mid p_l\right]\\&=p_l(1-p_l)+(1-2p_l)\mathbb{E}[\Delta p_l\mid p_l]-\mathrm{Var}\left(\Delta p_l\mid p_l\right)-\left(\mathbb{E}\left[\Delta p_l\mid p_l\right]\right)^2\\&=p_l(1-p_l)+(1-2p_l)\mathbb{E}[\Delta p_l\mid p_l]-\frac{p_l(1-p_l)}{2N_e}-\left(\mathbb{E}\left[\Delta p_l\mid p_l\right]\right)^2,\end{aligned}$$

and so,

$$\mathbb{E}\left[\Delta\{p_l(1-p_l)\}\right]=(1-2p_l)\mathbb{E}[\Delta p_l]-\left(\mathbb{E}\left[\Delta p_l\right]\right)^2-\frac{p_l(1-p_l)}{2N_e}. \tag{S.4}$$

Therefore, to estimate the expected change in the genic variance, we may substitute any expression for the expected change in allele frequencies at individual loci (e.g., Main Text Eqs. 5, 10, 25, or 34) into

$$\mathbb{E}\left[\Delta V_g\right]=\sum_l 2\alpha_l^2\left((1-2p_l)\mathbb{E}[\Delta p_l]-\left(\mathbb{E}\left[\Delta p_l\right]\right)^2-\frac{p_l(1-p_l)}{2N_e}\right). \tag{S.5}$$

Fig S4 shows that, when carrying out this procedure, our expressions for allele-frequency change (Eqs. 25 and 34) predict the change in the genic variance better than the classic expressions for allele-frequency change (Eqs. 5 and 10).

To calculate the change in the genetic variance, we use the expressions in Main Text Eq. (36) for the expected value of each pairwise LD coefficient $D_{ll'}^{(t)}$ between loci l and l' in generation t to estimate, in each generation, the expected reduction in the genetic variance owing to LD: $d=-2\sum_{l\neq l'}D_{ll'}^{(t)}\alpha_l\alpha_{l'}$. The expected change in the genetic variance $V_G=V_g-d$ is then

$$\mathbb{E}\left[\Delta V_G\right]=\mathbb{E}\left[\Delta V_g\right]-\mathbb{E}\left[\Delta d\right]. \tag{S.6}$$

S3 Mean phenotypes of a trait-increasing and trait-decreasing allele, and their subsequent frequency dynamics, when effect sizes and minor-allele frequencies differ across loci

The effect size and frequency of the trait-increasing allele at locus l are α_l and p_l. Let $v_l = 2p_l(1-p_l)\alpha_l^2$ be the contribution of locus l to the genic variance ($V_g = \sum_l v_l$), with $v_l \ll V_g$ for a highly polygenic trait.

Case 1: All loci are unlinked

We first consider the case where all loci are unlinked. The correlation coefficient between the allelic states at loci l and l' (polarized as being between the trait-increasing alleles at the two loci) is proportional to $\alpha_l \alpha_{l'}$, so that the expected LD between the loci $D_{ll'}$ is, in equilibrium, $-Kp_l(1-p_l)p_{l'}(1-p_{l'})\alpha_l\alpha_{l'}$ where K is a constant of proportionality [1,2]. As before, we denote the total reduction in the genetic variance due to the Bulmer effect by d, with this value specified by Eq. (17) in the Main Text. Then

$$\begin{aligned}
-d &= 2\sum_l \sum_{l' \neq l} D_{ll'}\alpha_l\alpha_{l'} \\
&= -2K\sum_l \sum_{l' \neq l} p_l(1-p_l)p_{l'}(1-p_{l'})\alpha_l^2\alpha_{l'}^2 \\
&= -K\sum_l p_l(1-p_l)\alpha_l^2 \sum_{l' \neq l} 2p_{l'}(1-p_{l'})\alpha_{l'}^2 \\
&= -K\sum_l p_l(1-p_l)\alpha_l^2(V_g - v_l) \\
&\approx -\frac{KV_g}{2}\sum_l 2p_l(1-p_l)\alpha_l^2 = -\frac{KV_g^2}{2}.
\end{aligned} \tag{S.7}$$

Therefore,

$$K \approx \frac{2d}{V_g^2}. \tag{S.8}$$

From Eq. (13), the mean phenotype experienced by the trait-increasing allele at locus l is

$$\begin{aligned}
\mathbb{E}[Y \mid I_l = 1] &= (1-p_l)\left(\alpha_l + \frac{1}{p_l(1-p_l)}\sum_{l' \neq l} D_{ll'}\alpha_{l'}\right) \\
&= (1-p_l)\left(\alpha_l - \frac{K}{p_l(1-p_l)}\sum_{l' \neq l} p_l(1-p_l)p_{l'}(1-p_{l'})\alpha_l\alpha_{l'}^2\right) \\
&= (1-p_l)\alpha_l\left(1 - \frac{K}{2}\sum_{l' \neq l} 2p_{l'}(1-p_{l'})\alpha_{l'}^2\right) \\
&\approx (1-p_l)\alpha_l\left(1 - \frac{d}{V_g^2}\cdot V_g\right) \\
&= (1-p_l)\alpha_l\left(1 - \frac{d}{V_g}\right).
\end{aligned} \tag{S.9}$$

Similarly, the mean phenotype experienced by the trait-decreasing allele at l is

$$\mathbb{E}[Y \mid I_l = 0] \approx -p_l \alpha_l \left(1 - \frac{d}{V_g}\right). \tag{S.10}$$

The 'effective' effect size of locus l is therefore

$$\alpha_{\text{eff}}^{(l)} \approx \alpha_l \left(1 - \frac{d}{V_g}\right), \tag{S.11}$$

and the change in frequency at the locus across a single generation is, in expectation,

$$\mathbb{E}\left[\Delta p_l\right] \approx p_l(1-p_l)\left(p_l - \frac{1}{2}\right)\frac{\left(\alpha_{\text{eff}}^{(l)}\right)^2}{V_S + V_P}, \tag{S.12}$$

as in the case where allele frequencies and effect sizes do not differ across loci.

Case 2: Linkage

If we allow for variable linkage relations among loci, then, in equilibrium, the expected LD between loci l and l' is $-Kp_l(1-p_l)p_{l'}(1-p_{l'})\alpha_l\alpha_{l'}/r_{ll'}$, where $r_{ll'}$ is the recombination fraction between loci l and l' and K is again a constant of proportionality [1,2]. If d is the total reduction in the genetic variance due to the Bulmer effect, with this value specified by Eq. (27) in the Main Text, then

$$\begin{aligned}
-d &= 2\sum_l \sum_{l' \neq l} D_{ll'}\alpha_l\alpha_{l'} \\
&= -2K \sum_l \sum_{l' \neq l} \frac{p_l(1-p_l)p_{l'}(1-p_{l'})\alpha_l^2\alpha_{l'}^2}{r_{ll'}} \\
&= -K \sum_l p_l(1-p_l)\alpha_l^2 \sum_{l' \neq l} \frac{2p_{l'}(1-p_{l'})\alpha_{l'}^2}{r_{ll'}} \\
&\approx -K \sum_l \frac{p_l(1-p_l)\alpha_l^2(V_g - v_l)}{r_{ll'}} \\
&\approx -\frac{KV_g}{2}\sum_l \frac{2p_l(1-p_l)\alpha_l^2}{r_{ll'}} \\
&\approx -\frac{KV_g^2}{2\bar{r}_h},
\end{aligned} \tag{S.13}$$

assuming independence of α_l and $\alpha_{l'}$ from $r_{ll'}$ and a weak dependence of p_l and $p_{l'}$ on $r_{ll'}$. Therefore, in this case,

$$K \approx \frac{2d\bar{r}_h}{V_g^2}. \tag{S.14}$$

From Eq. (13), the mean phenotype experienced by the trait-increasing allele at locus l is

$$\mathbb{E}[Y \mid I_l = 1] = (1-p_l)\left(\alpha_l + \frac{1}{p_l(1-p_l)}\sum_{l' \neq l} D_{ll'}\alpha_{l'}\right)$$

$$= (1-p_l)\left(\alpha_l - \frac{K}{p_l(1-p_l)}\sum_{l'\neq l} p_l(1-p_l)p_{l'}(1-p_{l'})\alpha_l\alpha_{l'}^2 \cdot \frac{1}{r_{ll'}}\right)$$

$$= (1-p_l)\alpha_l\left(1 - \frac{K}{2}\sum_{l'\neq l} 2p_{l'}(1-p_{l'})\alpha_{l'}^2 \cdot \frac{1}{r_{ll'}}\right)$$

$$\approx (1-p_l)\alpha_l\left(1 - \frac{d\bar{r}_h}{V_g^2}\cdot\frac{V_g}{\bar{r}_h^{(l)}}\right)$$

$$= (1-p_l)\alpha_l\left(1 - \frac{d}{V_g}\cdot\frac{\bar{r}_h}{\bar{r}_h^{(l)}}\right), \tag{S.15}$$

where the approximation follows from our assumption of a weak dependence of p_l and $p_{l'}$ on $r_{ll'}$. Similarly, the mean phenotype experienced by the trait-decreasing allele at l is

$$\mathbb{E}[Y \mid I_l = 0] \approx -p_l\alpha_l\left(1 - \frac{d}{V_g}\cdot\frac{\bar{r}_h}{\bar{r}_h^{(l)}}\right). \tag{S.16}$$

The 'effective' effect size of locus l is therefore

$$\alpha_{\text{eff}}^{(l)} \approx \alpha_l\left(1 - \frac{d}{V_g}\cdot\frac{\bar{r}_h}{\bar{r}_h^{(l)}}\right), \tag{S.17}$$

and the change in frequency at the locus across a single generation is, in expectation,

$$\mathbb{E}\left[\Delta p_l\right] = p_l(1-p_l)\left(p_l - \frac{1}{2}\right)\frac{\left(\alpha_{\text{eff}}^{(l)}\right)^2}{V_S + V_P}, \tag{S.18}$$

as in the case of equal effect sizes and minor-allele frequencies across loci.

References

[1] Lande R. The maintenance of genetic variability by mutation in a polygenic character with linked loci. Genetics Research. 1975;26(3):221–235.

[2] Bürger R. The Mathematical Theory of Selection, Recombination, and Mutation. New York: John Wiley & Sons; 2000.
