# Supplementary material for: The effect of long-range linkage disequilibrium on allele-frequency dynamics under stabilizing selection
Source: PLoS Genet. 2026 Mar 9;22(3):e1012035. doi: 10.1371/journal.pgen.1012035 (PMC12991805; doi:10.1371/journal.pgen.1012035)
Supplement: Attachment — Submitted filename: PG-D-25-01059-review.pdf [file pgen.1012035.s008.pdf]

Major general comments

This paper gives an analysis of the dynamics of allele frequency change at loci underlying a quantitative trait controlled by a large number of diallelic loci with small additive effects on the trait. This is equivalent to the famous "infinitesimal" model of Fisher 1918, although they do not use this term, nor do they cite Fisher. The trait is subject to the standard nor-optimal model of stabilizing selection, and it is assumed that the trait mean coincides with the optimum. From previous work on this problem, there is therefore an implicit assumption that variability is maintained by the balance between new mutations and selective elimination of alleles that either increase or decrease the trait value, depending on the state of the prevailing allele at a locus. However, mutation is not included in their mathematical modelling.

The novelty of the paper is that it provides results on the rates of change of allele frequencies that take into account the negative linkage disequilibrium (LD) among pairs of + (or – alleles) that is known to be generated by this form of stabilizing selection, the well-known"Bulmer effect". While previous work has provided formulae for the effect of LD on the net genetic variance of the trait, its effect on allele frequency change has been neglected, partly because the actual amount of LD between a given pair of loci is minute when trait is affected by many loci.

The authors have succeeded in extending the classic theory, and I do not question that their results are sound; they are basically a fairly straightforward and useful extension of earlier work. There is, however, an issue about the extent to which their findings substantially affect our understanding of selection on quantitative traits. They show figures (e.g. Figs. 1, 2) which suggest that there are noticeable differences in the rates of change of allele frequencies between formulae and simulations when LD is taken into account and those when it is ignored.

However, their model ignores mutation but assumes that the trait mean is at equilibrium. It's thus not clear to me how the perturbed allele frequencies shown in these figures fit into this framework. If a real population is truly at equilibrium under mutation and stabilizing selection, you won't see changes like this, except for effects of drift (which they don't model). If the population is not at such an equilibrium, it seems unlikely that the mean will coincide exactly with the optimum, in which case their results will not apply. I feel that they need to make a stronger case that their results have more than somewhat minor technical interest. For example, would the relation between effect size and equilibrium frequency under mutation-selection equilibrium be substantially different from the standard results? The pattern of increasing discrepancies as the allele frequencies decrease seen in the figures suggests that there could be a noticeable effect on the equilibrium under mutation and selection; it would be nice to know whether this is true.

A good deal of space could be saved by simply citing classic basic results rather than deriving them over again. There is nothing especially novel until l.229 (p.8), which is a long time for the reader to wait. Even here, the exposition is rather laboured, and could be more

concise. Their main result (eqns 22-24) can be derived more simply, as I show below in my specific comments.

Minor general comments

There are some notable omissions of references to relevant literature. For example, Wright (1935, J Genet 30:243, reprinted in Provine's selected papers volume) gave the first explicit treatment of selection against individual additive traits variants at loci underlying a trait subject to stabilizing selection and mutation, following on the more heuristic model of Fisher (1930 in The Genetical Theory of Natural Selection). The fundamental underdominant property of selection on rare variants was brought out in Wright's study, more than 20 years before Robertson (1956). They also do not cite Bürger's (2000) treatise on multilocus selection (see his Chapter 6), although admittedly it's difficult to see the wood for the trees in this book. There's also a treatment in Chapter 28 of the Walsh & Lynch (2018) tome. Of course, none of these workers analysed the effects of LD on the individual locus dynamics, but there is a clear implication that pairwise *D* values must be small unless linkage is very tight.

There are some small problems with the use of English that need correcting; I've flagged some of these, but got tired after a bit.

Specific comments

l.15 'of the type obtained.. ' not 'such as…'

l.22 'particularly' is redundant

l.30 As noted above, these results go back to Fisher and Wright.

l.31-38 This has been described repeatedly in the literature; is it necessary to go over it again?

l.37 'compared with' not 'to'

l.39 and elsewhere: no need for a hyphen in allele frequency

l.58 'found' not 'find'

l.59 'at' not 'in'

l.66 This the first mention of mutation; the core of the classical models of Fisher and Wright is the effect of mutation opposing the elimination of alleles by selection at nearly all the loci.

l.77 ‘of’ is redundant. This remark is at odds with their key assumption that the mean coincides with the optimum.

l.84 It should be mentioned that the loci are autosomal.

l.88 ‘alternative’ seems irrelevant

l.100 This was defined earlier as the ‘genic variance’.

l.114 There is an implicit assumption that mutation is unbiased with its effect on the trait; this has been relaxed in some studies (e.g. Charlesworth 2013 Genetics 194: 955); this leads to a departure of the equilibrium mean from the optimum and a directional selection component to the expression for allele frequency change. As a consequence, the selection coefficient on a variant is a linear rather than quadratic function of its effect, changing the dynamics considerably.

l.124 As noted above, Wright (1935) is a more appropriate citation; the l.131 eqn. comes from there. Space could be saved by simply citing his results; the rest of the material down to l.189 is fairly standard stuff.

l.129, 130 ‘A haploid instance..’ is an odd way of referring to a haplotype.

l.142-4 ‘inhabited by’ is also a strange term; ‘associated with’ is more usual.

l.147 I think we can do without this footnote. This is not at all new material.

l.178 ‘correcting for’ not ‘accounting for’ [which means ‘explain’].

l.189 If stabilizing selection is sufficiently weak, as is often assumed to be the case, $V_S \gg V_P$, so that the term V_P in can be ignored.

l.200-2 This repeats what has been said earlier, and is anyway not new.

l.210 Bulmer presents more material on this in his 1980 book.

l.229, 236 ‘in’ not ‘on’

l.232-251 I think the core result of eqns. 13/14 can be derived much more simply. The *D*’s are covariances of the indicator variable between the focal locus *l* and the *l*´ loci; the corresponding regression coefficient is thus the relevant *D* value divided by the variance $p_l(1 – p_l)$. The formula for the conditional expectation of *Y* follows from summing these terms and using eqn. 3. This is essentially the Price-Robertson formula for the correlated effects of selected loci on a focal locus

l.267 The corresponding quadratic equation for the general case is given by eqn. 9.47 of Bulmer's book; why do we need a reference to Veller & Coop? It seems pointless to go over the unlinked case in detail when the work has already been done for the more general case, where the harmonic mean of the recombination rates is included (obviously, this is just 0.5 with no linkage).

l.273 'do not'

l.287 This is just a special case of eqn. 9.46 in Bulmer's book.

l.296-305 These results seem to be very similar to eqn. 10.15 in Bulmer's book; I haven't had time to work through this, but the authors should check.

l.312 (and elsewhere) 'to' not 'of'

l.313 'from another' is redundant.

l.314-350 See my comment on l.296-305; the authors should go straight to the more general case.

l.316 There is a question of logic here; if the system is at equilibrium, why do the allele frequencies change?

l.390-405 Again, this seems to parallel the results on p.159 of Bulmer's book; the authors need to clarify whether there is really anything new here.

l.485-525 The treatment of LD caused by assortative mating is important for human applications, but the basic results are described in Crow & Kimura's 1970 textbook, pp.148-158, following on Fisher (1918). It seems strange not to cite these sources. In addition, it would probably be worth citing Yengo et al. (2018, Nature Hum. Behav. 2:948).

-
