# Supplementary material for: The effect of long-range linkage disequilibrium on allele-frequency dynamics under stabilizing selection
Source: PLoS Genet. 2026 Mar 9;22(3):e1012035. doi: 10.1371/journal.pgen.1012035 (PMC12991805; doi:10.1371/journal.pgen.1012035)
Supplement: Attachment — Submitted filename: NegmVeller_Responses.pdf [file pgen.1012035.s009.pdf]

Reviewer #1

In "The effect of long-range linkage disequilibrium on allele-frequency dynamics under stabilizing selection," Negm and Veller present a theoretical advancement for predicting allele frequency changes when those alleles contribute to stabilizing selection. Stabilizing selection is now understood to be important in explaining quantitative trait evolution and interpreting GWAS, and this work is a nice contribution towards gaining a population genetic perspective of common-place selection on complex traits. I believe it is sound and timely work, and I would be happy to see it published more-or-less as is.

We are grateful for the reviewer's positive assessment of our work.

I found no issues with the methods or theory, and the exposition is clear. I especially liked the discussion, which places their results in the broader context of other aspects affecting quantitative trait architectures, namely assortative mating and pleiotropy. While the paper lacks a direct application to empirical data, I don't believe it is warranted here as the theoretical advances help us in interpreting existing results in the literature and would be beyond the scope of this work.

The paper is somewhat technical, drawing from existing theory stretching back to the 1970s (Bulmer), but I think it is presented in a way that is not too unapproachable. For readers unfamiliar with this particular literature (which is deep and technical, as the authors point out), the paper could present a challenge. It's unavoidable that notation becomes a bit of a burden, so in editing, I would just suggest focusing on clarifying notation multiple times throughout, especially wrt Bulmer's original notation (d, X, etc). I think the authors already do a good job of this, really.

We have followed the reviewer's advice, adding reminders and clarifications of technical parameters in lines 286, 357, and 363. Additionally, in the spirit of the reviewer's comment, we have now made more explicit our discussion of the influence of assortative mating on an allele's "effective effect size" (lines 607–614).

My only other comment is about the choice of parameters in the simulations. The authors set $V_S/V_P = 5$, which I would have thought implies a whole lot of phenotypic variation relative to the strength of selection. If we take $V_G = 4UV_S$ (approx), this might imply a very high mutation rate and thus polygenicity. For height, as discussed on line 532, $V_S/V_P \sim 20–30$, and we think of height already as highly polygenic. A value of 5 might be unreasonably small, at least for interpreting human quantitative genetics results. My guess is that the authors chose such a value to amplify the effects they are modeling, which is understandable for a theory paper and demonstrating the accuracy of their results. But I could easily be wrong about this - justifying this choice of parameter values would be helpful.

We agree with the reviewer that our parameter choices involve quite strong selection. This is indeed to present a case where taking LD into account makes an especially noticeable difference—and, in a way, to "stress test" our calculations—although the value of V_S/V_P that we use is also consistent with the median quadratic selection gradient in the metanalysis of Kingsolver et al. (2001) among those indicative of stabilizing selection. We now note these rationales for our baseline parameter specification in lines 328–335.

We have also now checked our calculations against simulations with weaker selection, of a strength similar to that acting on human height, $V_S/V_P = 25$ (Stulp et al. 2012, Sanjak et al. 2017), and have shown that our calculations accurately predict allele-frequency dynamics in this case as well (Fig. S3). We are grateful to the reviewer for suggesting these additional analyses, which have helped to show the robustness of our results.

Reviewer #2

The manuscript by Negm and Veller uses analytical theory and simulations to study the impact of linkage disequilibrium on allele frequency changes under a model of stabilizing selection. They show that previous expressions under-estimate the change in allele frequencies. They also re-derive some well known results in an effort to reintroduce them to (human) geneticists. I found the manuscript to be well-written and the results interesting and I believe it is a good candidate for publication in PLOS Genetics. I have some suggestions below, but I do not think that they are necessary for publication.

We are very glad the reviewer found our work interesting, and are grateful for their positive assessment.

- Do the more accurate allele frequency dynamics do a better job of also predicting the change in additive genetic variance? If so, I think this would be sufficiently interesting to add to the main text.

This is an excellent suggestion. Our expressions for the expected change in allele frequencies, E[Δp_l], can be related to the expected change in the genic variance via the following expression, now derived in S1 Text Section S2:

$$\mathbb{E}[\Delta V_g] = \mathbb{E}\left[\Delta \sum_l 2p_l(1-p_l)\alpha_l^2\right] = \sum_l 2\alpha_l^2 \left\{(1-2p)\mathbb{E}\left[\Delta p_l\right] - \left(\mathbb{E}\left[\Delta p_l\right]\right)^2 - \frac{p_l(1-p_l)}{2N_e}\right\}.$$

Substituting our formula for E[Δp_l] into the expression above accurately predicts the change in the genic variance observed in simulations, whereas substituting the classic formulae for E[Δp_l] does not, as we now show in Fig. S4. Furthermore, taking into account the expressions for the buildup of LD under stabilizing selection (Eq. 36), we can also use the above expression to predict the change in the genetic variance over time, as shown in Fig. S4. We are grateful to the reviewer for this suggestion, which has led to an important extension of our results.

- I am curious about the impact of demographic history on these results (particularly: bottlenecks, population growth, population structure+migration). I understand that carrying out simulations to explore this would be a large undertaking. Instead, I wondered if the authors could add a discussion of the expected impact of demography on their results. This would help connect the discussion on polygenic risk score portability.

We have now included a discussion of the robustness of our results to demography in a new Discussion subsection, “Assumptions, and notions of equilibrium under stabilizing selection” (lines 524–572). There, we note that our calculations are robust to changes in demography, because they rely only on the assumptions that the trait mean is at its optimum and that the LD generated by stabilizing selection is at its quasi-equilibrium value. The expected LD generated by selection (and the rate at which it is generated) is independent of population size and structure, and, except in extremely small populations, the trait mean will tend to be very close to its optimum.

- In the discussion of allelic turnover, I thought the authors may want to cite a couple of additional papers that touch on this point: PMID: 31201529, PMID: 27197206, PMID: 33691092.

We agree that these are relevant references and have added them in.

- In addition, in the discussion of signed LD, a recent study has looked at this empirically in the UK Biobank: PMID: 38106023.

We have now included this reference.

- The discussion of pleiotropy is very interesting and I wonder if the model gives the authors an opportunity to set some bounds based on LD about the total number of independent traits that could be under selection?

This is a very interesting idea, but given that the degree and nature of pleiotropy in complex traits is a topic of major current focus in the field, we are hesitant to make quantitative claims without extensive additional analyses to back them up, and leave this for future work.

Reviewer #3

Major general comments

This paper gives an analysis of the dynamics of allele frequency change at loci underlying a quantitative trait controlled by a large number of diallelic loci with small additive effects on the trait. This is equivalent to the famous "infinitesimal" model of Fisher 1918, although they do not use this term, nor do they cite Fisher. The trait is subject to the standard nor-optimal model of stabilizing selection, and it is assumed that the trait mean coincides with the optimum. From previous work on this problem, there is therefore an implicit assumption that variability is maintained by the balance between new mutations and selective elimination of alleles that either increase or decrease the trait value, depending on the state of the prevailing allele at a locus. However, mutation is not included in their mathematical modelling.

The novelty of the paper is that it provides results on the rates of change of allele frequencies that take into account the negative linkage disequilibrium (LD) among pairs of + (or – alleles) that is known to be generated by this form of stabilizing selection, the well- known "Bulmer effect". While previous work has provided formulae for the effect of LD on the net genetic variance of the trait, its effect on allele frequency change has been neglected, partly because the actual amount of LD between a given pair of loci is minute when the trait is affected by many loci.

The authors have succeeded in extending the classic theory, and I do not question that their results are sound; they are basically a fairly straightforward and useful extension of earlier work. There is, however, an issue about the extent to which their findings substantially affect our understanding of selection on quantitative traits. They show figures (e.g. Figs. 1, 2) which suggest that there are noticeable differences in the rates of change of allele frequencies between formulae and simulations when LD is taken into account and those when it is ignored.

We are glad that the reviewer found that the original results in our manuscript are novel, and also for their assessment that these results will be useful.

However, their model ignores mutation but assumes that the trait mean is at equilibrium. It's thus not clear to me how the perturbed allele frequencies shown in these figures fit into this framework. If a real population is truly at equilibrium under mutation and stabilizing selection, you won't see changes like this, except for effects of drift (which they don't model). If the population is not at such an equilibrium, it seems unlikely that the mean will coincide exactly with the optimum, in which case their results will not apply.

The reviewer raises several issues here, and we respond to each in turn below. First, though, we note that the reviewer's comments conflate several notions of "equilibrium" under stabilizing selection. Although we do not assume equilibrium in our calculations—indeed, this is one of their strengths—the simplest

definition is that the trait distribution is stationary. We have now clarified this in a subsection of the Discussion (lines 524–572).

(1) However, their model ignores mutation but assumes that the trait mean is at equilibrium.
We do not assume equilibrium in our calculations. We do assume that the trait mean is at its optimum, because this will happen very quickly even if the trait is initially displaced from its optimum, and we are interested in the genetic dynamics after this directional phase of selection, once stabilizing selection commences. We have clarified this in lines 118–121. Note that assuming the mean is at its optimum is not the same as assuming that the trait is at equilibrium, because the trait's variance equilibrates on a much longer timescale than its mean—we have now emphasized this in the Discussion (lines 529–531).

(2) It's thus not clear to me how the perturbed allele frequencies shown in these figures fit into this framework. If a real population is truly at equilibrium under mutation and stabilizing selection, you won't see changes like this, except for effects of drift (which they don't model).
The reviewer implies that, at equilibrium under stabilizing selection, allele frequencies would be constant. But this is never true for complex traits, the mutational targets of which are much larger than the number of polymorphic loci that contribute variation at any point in time (e.g., $\mathcal{O}(10^7)$ vs. $\mathcal{O}(10^4)$ for human height; Simons et al. 2025, Yengo et al. 2022). For such traits, the vast majority of new trait-affecting mutations do not occur at currently polymorphic loci; the frequency dynamics at these loci are therefore dictated by selection, not mutation, and are thus well approximated by the equations that we have derived. We have now clarified this in lines 548–555 of the Discussion.

(3) If the population is not at such an equilibrium, it seems unlikely that the mean will coincide exactly with the optimum, in which case their results will not apply.
As noted above, the mean trait value will rapidly move to its optimum, and will do so long before the variance has equilibrated. So it is natural to assume that the mean is at its optimum even if one does not assume equilibrium. This is noted in lines 529–531 and 541–542.

I feel that they need to make a stronger case that their results have more than somewhat minor technical interest. For example, would the relation between effect size and equilibrium frequency under mutation-selection equilibrium be substantially different from the standard results? The pattern of increasing discrepancies as the allele frequencies decrease seen in the figures suggests that there could be a noticeable effect on the equilibrium under mutation and selection; it would be nice to know whether this is true.

As we have shown, the dynamics—in and out of equilibrium—are well approximated by replacing the effect size of each allele with an "effective effect size", reduced from the true effect size by a factor that we have calculated. This factor is the source of the differences between the trajectories in our figures, and these differences can be seen to be substantial, including under weaker selection (Fig. S3). While our predicted trajectories, in some cases (e.g., Fig. 2B), do not perfectly predict the simulated trajectories, they are nonetheless a very good fit, and much better than the classical predictions. Moreover, in other cases (e.g., Figs. 1 and 2A), our predictions are essentially a perfect fit to the simulated trajectories.

A good deal of space could be saved by simply citing classic basic results rather than deriving them over again. There is nothing especially novel until l.229 (p.8), which is a long time for the reader to wait. Even here, the exposition is rather laboured, and could be more concise. Their main result (eqns 22-24) can be derived more simply, as I show below in my specific comments.

Our intended audience includes empirical practitioners who might not be familiar with the treatment of these classic results in Bulmer's, Bürger's, and Lynch & Walsh's very technical books, and who might benefit from a simpler, more intuitive derivation. We note that the other reviewers and the academic

editor see value in our presentation of these results and—subject to editorial discretion—we would prefer to maintain this presentation.

Minor general comments

There are some notable omissions of references to relevant literature. For example, Wright (1935, J Genet 30:243, reprinted in Provine's selected papers volume) gave the first explicit treatment of selection against individual additive traits variants at loci underlying a trait subject to stabilizing selection and mutation, following on the more heuristic model of Fisher (1930 in The Genetical Theory of Natural Selection). The fundamental underdominant property of selection on rare variants was brought out in Wright's study, more than 20 years before Robertson (1956). They also do not cite Bürger's (2000) treatise on multilocus selection (see his Chapter 6), although admittedly it's difficult to see the wood for the trees in this book. There's also a treatment in Chapter 28 of the Walsh & Lynch (2018) tome. Of course, none of these workers analysed the effects of LD on the individual locus dynamics, but there is a clear implication that pairwise D values must be small unless linkage is very tight.

We have now cited Wright 1935 alongside Robertson 1956 for the classic "underdominance" equation, and have furthermore cited the books of Bürger, Walsh & Lynch, and Bulmer books in the Introduction.

There are some small problems with the use of English that need correcting; I've flagged some of these, but got tired after a bit.

Specific comments

l.15 'of the type obtained.. ' not 'such as...'

We have changed "such as from" to "such as those from".

l.22 'particularly' is redundant

removed

l.30 As noted above, these results go back to Fisher and Wright.

We have now cited Wright 1935 alongside Robertson 1956 here.

l.31-38 This has been described repeatedly in the literature; is it necessary to go over it again?

These lines give a simple but mathematically correct verbal intuition for why, under stabilizing selection, the frequency dynamics at individual loci resemble underdominant selection. We have found, in discussing stabilizing selection with students and colleagues, that there is often confusion on this point, and so, again with an eye towards readers who are not very familiar with the technical literature on stabilizing selection, we would prefer to keep this intuition in.

l.37 'compared with' not 'to'

These have different meanings in this context: "maladapted to the rest of the genome" vs. "maladapted compared with the rest of the genome". Our original has the meaning we intended.

l.39 and elsewhere: no need for a hyphen in allele frequency

The relevant phrase is “allele-frequency change”, in which “allele” and “frequency” form a compound adjective which we believe should be hyphenated.

l.58 ‘found’ not ‘find’

changed

l.59 ‘at’ not ‘in’

changed

l.66 This the first mention of mutation; the core of the classical models of Fisher and Wright is the effect of mutation opposing the elimination of alleles by selection at nearly all the loci.

As noted above, for complex traits with extremely large mutational targets (larger than could have been envisaged by Fisher and Wright), most new mutations will occur at loci that are not currently contributing substantial variance; consequently, the frequency dynamics at these latter loci will be largely unaffected by mutation. We have now clarified this in lines 548–555.

l.77 ‘of’ is redundant. This remark is at odds with their key assumption that the mean coincides with the optimum.

We have removed “of”. The remark in question is that we are interested in the genetic dynamics of stabilizing selection in out-of-equilibrium scenarios. As noted above, assuming that the mean trait value is at its optimum is different from and much less restrictive than assuming trait equilibrium. We have now discussed this point in lines 529–531 and 541–542.

l.84 It should be mentioned that the loci are autosomal.

done

l.88 ‘alternative’ seems irrelevant

removed

l.100 This was defined earlier as the ‘genic variance’.

In the spirit of Reviewer 1’s helpful comments, we prefer to restate the distinction between the genetic and genic variances here in the Model section.

l.114 There is an implicit assumption that mutation is unbiased with its effect on the trait; this has been relaxed in some studies (e.g. Charlesworth 2013 Genetics 194: 955); this leads to a departure of the equilibrium mean from the optimum and a directional selection component to the expression for allele frequency change. As a consequence, the selection coefficient on a variant is a linear rather than quadratic function of its effect, changing the dynamics considerably.

We have now noted this in lines 555–558.

l.124 As noted above, Wright (1935) is a more appropriate citation; the l.131 eqn. comes from there. Space could be saved by simply citing his results; the rest of the material down to l.189 is fairly standard stuff.

We have now cited Wright 1935 alongside Robertson 1956. As noted above, we prefer to keep our full treatment, subject to editorial discretion.

l.129, 130 ‘A haploid instance..’ is an odd way of referring to a haplotype.

The reason we chose the admittedly clunky “haploid instance” over the simpler “haplotype” is that “haplotype” might invoke multiple linked loci, which we do not intend here.

l.142-4 ‘inhabited by’ is also a strange term; ‘associated with’ is more usual.

We prefer the more direct “inhabited by”.

l.147 I think we can do without this footnote. This is not at all new material.

The footnote shows that the heterozygous genotype is never the least fit under stabilizing selection (and references Robertson 1956). We have found that this is a point of confusion for many in understanding the underdominant-like dynamics of stabilizing selection, and would therefore prefer to keep it in.

l.178 ‘correcting for’ not ‘accounting for’ [which means ‘explain’].

Changed to “taking into account”.

l.189 If stabilizing selection is sufficiently weak, as is often assumed to be the case, $V_S >> V_P$, so that the term V_P in can be ignored.

Ignoring V_P in Eq. (10) returns Eq. (5), and the predictions of the two equations can be meaningfully different, including for selection of a strength estimated for some human traits (compare pink and yellow lines in Figs. 1, 2, S3).

l.200-2 This repeats what has been said earlier, and is anyway not new.

In the spirit of Reviewer 1’s helpful comments, we prefer to keep this reminder in.

l.210 Bulmer presents more material on this in his 1980 book.

We have now additionally cited Bulmer’s book here alongside his 1971 and 1974 papers.

l.229, 236 ‘in’ not ‘on’

done

l.232-251 I think the core result of eqns. 13/14 can be derived much more simply. The *D*’s are covariances of the indicator variable between the focal locus l and the l' loci; the corresponding regression coefficient is thus the relevant *D* value divided by the variance $p_l(1 - p_l)$. The formula for the conditional expectation of *Y* follows from summing these terms and using eqn. 3. This is essentially the Price-Robertson formula for the correlated effects of selected loci on a focal locus.

We consider our approach more direct, but have now also outlined the reviewer’s alternative approach in lines 314–321, in case some readers find it more intuitive.

l.267 The corresponding quadratic equation for the general case is given by eqn. 9.47 of Bulmer's book; why do we need a reference to Veller & Coop? It seems pointless to go over the unlinked case in detail when the work has already been done for the more general case, where the harmonic mean of the recombination rates is included (obviously, this is just 0.5 with no linkage).

We had previously cited Bulmer 1971 & 1974 for the quadratic equations (which are reproduced with slightly different notation in his 1980 book) and Veller & Coop 2024 for reporting which roots of these equations are the relevant ones (this is surprisingly nontrivial—see SI pgs. 29 and 30 in Veller & Coop). On the ordering of our results, we prefer—again, for a broader readership—to build up from the simpler case of no linkage to the more complicated case of linkage, though the latter of course nests the former.

l.273 'do not'

done

l.287 This is just a special case of eqn. 9.46 in Bulmer's book.

Bulmer's Eq. 9.46 gives the aggregate LD, summed across all pairs of loci. The simple point we make here is that, when all loci are unlinked, this aggregate LD is apportioned equally in expectation to each pair of loci. This gives the expected individual-locus-pair LD values, which are required for our calculations.

l.296-305 These results seem to be very similar to eqn. 10.15 in Bulmer's book; I haven't had time to work through this, but the authors should check.

We are grateful to the reviewer for directing us to this chapter of Bulmer's book. There, Bulmer applies an approach similar to the Price–Robertson approach suggested above by the reviewer and obtains an expression, though without spelling it out, that can be translated to our Eq. (25). We have now cited Bulmer for this after outlining the reviewer's Price–Robertson approach in lines 314–321. We note that this does not apply to our more general expression, Eq. (46), that takes into account the linkage relations of individual loci.

l.312 (and elsewhere) 'to' not 'of'

done

l.313 'from another' is redundant.

removed

l.314-350 See my comment on l.296-305; the authors should go straight to the more general case.

As noted above, with an eye toward readers unfamiliar with the theoretical literature on stabilizing selection, we prefer to treat the simpler case of no linkage first before building up to the more complicated case of linkage.

l.316 There is a question of logic here; if the system is at equilibrium, why do the allele frequencies change?

As noted above, we do not assume that the system is at equilibrium, and as clarified in the Discussion, allele frequencies do change, even at equilibrium, for complex traits with large mutational targets.

l.390-405 Again, this seems to parallel the results on p.159 of Bulmer's book; the authors need to clarify whether there is really anything new here.

We had cited Bulmer 1974 in this section, and have now additionally cited his 1980 book.

l.485-525 The treatment of LD caused by assortative mating is important for human applications, but the basic results are described in Crow & Kimura's 1970 textbook, pp.148-158, following on Fisher (1918). It seems strange not to cite these sources. In addition, it would probably be worth citing Yengo et al. (2018, Nature Hum. Behav. 2:948).

We had previously cited Crow & Felsenstein 1968 (on which the Crow & Kimura treatment is based) and Wright 1921 (which we think is a more explicit treatment of assortative mating than Fisher 1918). We have now additionally cited Yengo 2018.
